# Effects of medical insurance system on the hospitalization cost of acute myocardial infarction patients

**DOI:** 10.1186/s12962-022-00343-6

**Published:** 2022-02-22

**Authors:** Ying-Hong Chu, Gui-Hua Jiang, Hong Zhang, Xiao-Rong Luan

**Affiliations:** 1 Key Laboratory of Cardiovascular Proteomics of Shandong Province, Department of Geriatric Medicine, Qilu Hospital of Shandong University, Ji’nan, 250012 People’s Republic of China; 2 Key Laboratory of Cardiovascular Remodeling and Function Research Chinese Ministry of Education and Chinese Ministry of Public Health, Department of Cardiology, Qilu Hospital of Shandong University, Ji’nan, 250012 People’s Republic of China; 3 Nursing Department of Qilu Hospital of Shandong University, Ji’nan, 250012 People’s Republic of China

**Keywords:** Acute myocardial infarction, Health insurance status, Cost effectiveness, Nursing

## Abstract

**Background:**

Acute myocardial infarction is still a burden on Chinese patients. Whether different medical insurance system have any influence on the hospitalization cost and therapeutic effect of acute myocardial infarction patient needs further investigation.

**Method:**

In this study, 600 patients were stratified by health insurance status to investigate the cost effectiveness.

**Result:**

Compared with free medical care, patients with other health insurance status have a significantly lower age (*P* ˂ 0.05–0.001), the youngest of which is new rural cooperative medical system. The hospital expense, nursing fee, length of stay, daily hospitalization cost, daily drug cost, daily nursing cost and percent of nursing cost of different health insurance status were statistically significant. ANCOVA analyses controlling for age showed that the differences of hospital expenses, nursing fee, length of stay and daily hospitalization cost were still statistically significant. Further studies found that health insurance status was the leading factors influencing length of stay (β = − 0.305, *P* = 0.0000001), nursing costs (β = − 0.319, *P* = 0.004), daily hospitalization costs (β = 0.296, *P* = 0.0001) and occurrence of clinical events (β = − 0.186, OR = 0.830, 95% CI 0.694–0.993, *P* = 0.041).

**Conclusions:**

The hospitalization cost, length of stay, nursing work and therapeutic effect of acute myocardial infarction patients are affected by different health insurance status and age.

## Background

Cardiovascular disease is the first major disease threatening human health, and the cost of cardiovascular disease management and intervention is high, accounting for 25% of the total medical cost [1]. Acute myocardial infarction, due to the increasing incidence, high medical costs and rapid growth, is still a burden on Chinese patients [3]. Moreover, the hidden expenditure of family nursing and missed work caused by hospitalization further aggravates the family burden of patients with acute myocardial infarction. Since 2004, the total hospitalization cost of acute myocardial infarction in China has increased at an average annual rate of 29.15%. The average hospitalization cost of acute myocardial infarction in 2016 was about ¥26,000, the average annual growth rate of which has been 7.12% [2]. Although the number of insured people in China has reached 1.295 billion, universal medical insurance is not equal to comprehensive medical insurance. Actually, the reimbursement for AMI related hospitalization varies by different types of insurance.

At present, China’s medical insurance system is diverse, including free medical care, provincial medical insurance, municipal urban employee medical insurance, municipal urban resident medical insurance, new rural cooperative medical system, self-payment and other categories. Different types of insurance systems cover different population based on their residence in urban or rural, the nature of their occupations and different length of employment. Different types of insurance systems mean that patients afford their care differently. Usually, those with free medical care would absorb no medical cost, while those with provincial medical insurance, municipal urban employee medical insurance, municipal urban resident medical insurance and new rural cooperative medical system would afford more and more accordingly, and those with self-payment would themselves bear all the medical cost completely. According to the REACH study, countries with higher own burden medical expenses are more likely to have persistent total cholesterol rise [4]. So in China, does different medical insurance system have any influence on the hospitalization cost and therapeutic effect of acute myocardial infarction patients? There is no relevant research yet.

For a long time, our research has focused on the treatments, such as drugs and surgery, total cost and therapeutic effect. Few studies have dealt with the therapeutic effect of nursing on diseases. The just nursing researches mostly followed clinical nursing pathway research [5], almost abandoning the actual nursing and medical intervention. However, in patients with acute myocardial infarction, medical treatment and nursing care cannot be separated as a whole. In this whole, does nursing care have any influence on hospitalization cost and therapeutic effect? It warrants further investigation.

## Methods

### Ethics statement

This study was approved by the ethics committee of Qilu Hospital of Shandong University. Written informed consent was acquired from the participants for their clinical records to be used in this study. All data were anonymized and de-identified prior to analysis.

### Case file collection

The case files of consecutive patients were identified with a discharge diagnosis of AMI at Qilu Hospital of Shandong University between January 2011 and May 2013. AMI were defined according to the European Society of Cardiology guidelines for the management of AMI [6]. Inclusion criteria were discharge diagnosis of AMI, age ≥ 20 years, hospitalization for 48 h or more solely due to AMI, with complete insurance information, from January 1, 2011 to May 31, 2013. Exclusion criteria were age < 20 years old, duplicate data, with mental and cognitive illness diagnosis in the past or with incomplete insurance information. In total, 600 patients were included. Information taken from these case files included sex, diagnosis on admission, discharge diagnosis, length of stay, hospital costs, laboratory examination, complications (including acute heart failure, atrial arrhythmia, ventricular arrhythmia and death), and drug treatment.

### Grouping

Patients were stratified by health insurance status, namely, free medical care (n = 27), provincial medical insurance (n = 26), municipal urban employee medical insurance (n = 216), municipal urban resident medical insurance (n = 57), new rural cooperative medical system (n = 150), and self-payment (n = 124).

### Statistical analysis

All analyses were conducted using Statistical Package for the Social Sciences version 18.0 software (SPSS Inc., Chicago, IL, USA) and *P* ˂ 0.05 was considered to be statistically significant. The results are expressed as the $\overline{x}$±*s* or as a proportion (%). For categorical variables, the χ^2^ test was used. Analysis of variance was used to study the impact of health insurance status on duration of length of stay and costs. Analysis of Covariance (ANCOVA) was used to detect associations and produce adjusted means for age. Multiple linear regressions using the stepwise method were performed to examine predictors of hospital costs. Binary logistic regressions using the forward conditional method were performed to examine individual predictors of clinical cardiovascular events.

## Results

### Comparison of baseline characteristics

Compared with free medical care (mean 76 years old), patients with other health insurance status have a significantly lower age (mean 59–69 years old, *P* ˂ 0.05–0.001, Table 1), the youngest of which is new rural cooperative medical system (mean 59 years old). The gender proportion was similar in all groups except for municipal urban resident medical insurance (more females) and self-payment (more males). Although there were significantly differences in Cr (creatinine) and CysC (Cystatin C), which of new rural cooperative medical system (73.03 ± 49.58, 1.01 ± 0.39, correspondingly, Table 1) was low, there was no statistically significant difference in ANCOVA analysis after controlling for age. ANCOVA analyses of CK-MB (creatine kinase isoenzyme) and cTnI (cardiac troponin I) controlling for age showed no statistically significant difference. These results suggest that there is no difference in baseline disease status among hospitalized patients despite differences in age.

**Table 1:** Comparison of baseline characteristics in different health insurance status

	Free medical care (n = 27)	Provincial medical insurance (n = 26)	Municipal urban employee medical insurance (n = 216)	Municipal urban resident medical insurance (n = 57)	New rural cooperative medical system (n = 150)	Self-payment (n = 124)
Age (y)	76 ± 11	69 ± 13*	66 ± 13***	68 ± 14**	59 ± 12***^††‡‡‡§§§^	64 ± 13***^§║║^
Sex (M/F)	18/9	17/9	153/63	28/29^‡^	100/50	97/27^§§^
HR (bpm)	73 ± 9	79 ± 16	78 ± 16	82 ± 16	76 ± 15	75 ± 15
SBP (mmHg)	133 ± 24	136 ± 23	130 ± 22	138 ± 24	130 ± 21	132 ± 22
DBP (mmHg)	69 ± 10	77 ± 14*	75 ± 12*	78 ± 14**	77 ± 13**	76 ± 12*
Hb (g/L)	131.88 ± 16.22	131.35 ± 22.44	129.48 ± 28.54	123.15 ± 22.19	132.70 ± 27.89	129.05 ± 32.20
PLT (× 10^9^/L)	184.33 ± 63.53	205.92 ± 62.63	201.68 ± 92.37	204.67 ± 65.09	207.82 ± 73.15	194.93 ± 73.97
ALT (U/L)	25.74 ± 16.08	44.81 ± 68.31	39.70 ± 61.80	30.74 ± 28.83	46.51 ± 54.23**	32.27 ± 28.40^║^
AST (U/L)	65.44 ± 77.23	86.82 ± 102.18	82.31 ± 143.35	66.88 ± 73.29	64.92 ± 79.82	67.42 ± 94.63
TC (mmol/L)	4.52 ± 0.89	4.66 ± 1.07	4.55 ± 1.08	4.50 ± 1.54	4.32 ± 1.22	4.52 ± 1.24
TG (mmol/L)	1.23 ± 0.70	1.76 ± 1.98	1.43 ± 0.73	1.49 ± 1.28	1.55 ± 0.85	1.50 ± 0.79
HDL-C (mmol/L)	1.17 ± 0.28	1.05 ± 0.21	1.11 ± 0.26	1.10 ± 0.22	1.09 ± 0.28	1.08 ± 0.25
LDL-C (mmol/L)	2.85 ± 0.75	3.08 ± 0.87	2.88 ± 0.92	2.86 ± 1.07	2.65 ± 0.96	2.85 ± 1.02
BS (mmol/L)	5.64 ± 1.89	6.30 ± 2.65	6.43 ± 2.51	6.65 ± 2.97	6.14 ± 2.48	6.67 ± 2.56
BUN (mmol/L)	5.82 ± 2.65	6.08 ± 3.02	5.58 ± 2.82	6.33 ± 2.47	5.53 ± 4.53	5.41 ± 2.85
Cr (µmol/L)	80.26 ± 23.48	75.12 ± 25.19	81.26 ± 38.14	70.98 ± 22.28	73.03 ± 49.58^‡‡^	79.51 ± 43.75
CysC (mg/L)	1.24 ± 0.47	1.05 ± 0.26	1.14 ± 0.39	1.07 ± 0.28	1.01 ± 0.39**^‡‡^	1.10 ± 0.53
K (mmol/L)	4.04 ± 0.32	4.03 ± 0.43	4.05 ± 0.50	4.06 ± 0.60	4.09 ± 0.44	4.04 ± 0.65
Na (mmol/L)	139.44 ± 4.34	138.65 ± 4.08	139.65 ± 3.68	138.09 ± 4.19	139.36 ± 3.34	139.32 ± 4.22
CK (U/L)	555.55 ± 883.41	562.05 ± 773.67	431.16 ± 894.77	300.64 ± 467.53	250.79 ± 482.00^†^	288.28 ± 545.29
CK-MB (ng/mL)	44.31 ± 101.47	38.72 ± 64.65	31.06 ± 75.63	22.98 ± 40.22	19.07 ± 63.84*^†‡§^	24.94 ± 56.55
cTnI (ng/L)	17.18 ± 51.89	14.53 ± 34.32	12.78 ± 31.29	12.42 ± 27.27	11.16 ± 27.34	7.33 ± 18.41

*HR* heart rate, *SBP* systolic blood pressure, *DBP* diastolic blood pressure, *Hb* hemoglobin, *PLT* platelet, *ALT* alanine aminotransferase, *AST* aspartate aminotransferase, *TC* total cholesterol, *TG* triglyceride, *HDL-C* high density lipoprotein cholesterol, *LDL-C* low density lipoprotein cholesterol, *BS* blood sugar, *BUN* blood urea nitrogen, *Cr* creatinine, *CysC* cystatin C, *CK* creatine kinase, *CK-MB* creatine kinase isoenzyme, *cTnI* cardiac troponin I

Compared with free medical care, *P < 0.05, **P < 0.01, ***P < 0.001; compared with provincial medical insurance, ^†^*P* < 0.05, ^††^*P* < 0.01, ^†††^*P* < 0.001; compared with municipal urban employee medical insurance, ^‡^*P* < 0.05, ^‡‡^*P* < 0.01, ^‡‡‡^*P* < 0.001; compared with municipal urban resident medical insurance, ^§^*P* < 0.05, ^§§^*P* < 0.01, ^§§§^*P* < 0.001; compared with new rural cooperative medical system, ^║^*P* < 0.05, ^║║^*P* < 0.01, ^║║║^*P* < 0.001

### Comparison of hospitalization costs of patients with different health insurance status

The difference in hospital expenses of different health insurance status was statistically significant, among which free medical care was the largest (6.40 ± 3.05, Table 2). ANCOVA analyses controlling for age showed that the difference was still statistically significant, suggesting that age was not the main factor determining the cost, and there were differences in hospital expenses of different health insurance status. However, statistical differences still existed among different health insurance status after controlling for age.

**Table 2:** Comparison of hospitalization costs of patients with different health insurance status

	Free medical care (n = 27)	Provincial medical insurance (n = 26)	Municipal urban employee medical insurance (n = 216)	Municipal urban resident medical insurance (n = 57)	New rural cooperative medical system (n = 150)	Self-payment (n = 124)
Total cost (×10^4^)	6.40 ± 3.05	4.10 ± 3.17**	4.06 ± 2.65***	4.02 ± 2.94***	4.59 ± 2.50**	4.85 ± 2.89**^‡^
Medication cost (×10^4^)	2.55 ± 1.34	1.54 ± 1.04***	1.25 ± 0.76***	1.28 ± 0.72***	0.93 ± 0.53***^††‡‡‡§§^	1.38 ± 1.09***^║║║^
Nursing cost	377.11 ± 949.17	112.92 ± 57.50**	160.18 ± 332.69***	137.28 ± 114.02***	94.03 ± 104.90***‡	119.59 ± 178.74***
Length of stay (d)	18.63 ± 7.42	13.50 ± 6.29***	13.03 ± 5.92***	12.86 ± 4.65***	9.59 ± 3.85***^†††‡‡‡§§§^	11.40 ± 5.96***^‡‡║║^
Daily cost (×10^3^)	3.69 ± 1.88	3.74 ± 3.77	3.44 ± 2.71	3.10 ± 2.08	5.39 ± 3.72*^†‡‡‡§§§^	5.08 ± 4.06*^‡‡‡§§§^
Daily medication cost (×10^3^)	1.40 ± 0.67	1.16 ± 0.42	0.96 ± 0.38***	0.97 ± 0.35***	1.00 ± 0.53***	1.20 ± 0.55*^‡‡‡§§║║^
Daily nursing cost	17.28 ± 36.20	8.33 ± 1.58**	10.44 ± 9.89**	10.51 ± 7.18*	9.49 ± 7.69**	9.84 ± 8.67**
Percent of nursing cost (×10^–3^)	5.25 ± 9.25	4.03 ± 2.63	4.69 ± 4.72	4.70 ± 2.71	3.29 ± 4.95*^‡‡^	3.40 ± 3.61^‡^

Compared with free medical care, *P < 0.05, **P < 0.01, ***P < 0.001; compared with provincial medical insurance, ^†^*P* < 0.05, ^††^*P* < 0.01, ^†††^*P* < 0.001; compared with municipal urban employee medical insurance, ^‡^*P* < 0.05, ^‡‡^*P* < 0.01, ^‡‡‡^*P* < 0.001; compared with municipal urban resident medical insurance, ^§^*P* < 0.05, ^§§^*P* < 0.01, ^§§§^*P* < 0.001; compared with new rural cooperative medical system, ^║^*P* < 0.05, ^║║^*P* < 0.01, ^║║║^*P* < 0.001

Nursing cost is theoretically related to the increase of age. The older the patient (76 ± 11 years old in free medical care group) is, the more dependent on nursing fee (377.11 ± 949.17 in free medical care group) is. However, there is no statistical significance in the influence of age on nursing fee among AMI patients, and the difference of nursing fee between health insurance status has statistical significance, and the nursing fee of new rural cooperative medical system is low (Table 2).

The difference in length of stay among different health insurance status was statistically significant, among which the maximum length of stay was at free medical care (18.63 ± 7.42) and the shortest was at the new rural cooperative medical system (9.59 ± 3.85, Table 2). ANCOVA analysis controlling the age showed that the difference was still statistically significant, suggesting that age was not the main factor determining the length of stay, and there were differences in length of stay among different health insurance status. The difference of daily hospitalization cost between health insurance status was statistically significant, and the daily hospitalization cost of the new rural cooperative medical system (5.39 ± 3.72, Table 2) was the highest. After controlling for age, ANCOVA analysis showed that the difference was still statistically significant, suggesting that age was not the main factor determining the cost.

The daily drug cost is theoretically related to the increase of age and comorbidity. However, there is no statistical significance in the influence of age on daily drug cost among AMI patients, and the difference of daily drug cost between health insurance status is statistically significant, and the daily drug cost at free medical care (1.40 ± 0.67, Table 2) is highest. The daily nursing cost was the highest at free medical care (17.28 ± 36.20, Table 2), but ANCOVA analysis after controlling for age showed no statistically significant differences in age and daily nursing cost, suggesting no impact of age. The percent of nursing cost was statistically significant among different health insurance status, and the percent in new rural cooperative medical system (3.29 ± 4.95, Table 2) was the smallest. However, ANCOVA analysis after controlling for age showed no statistical significance in the percent of nursing cost, suggesting which was age-related.

### Analyses of relevant factors

Further studies were conducted on the factors influencing the total cost of hospitalization, and blood potassium (β = 0.367, *P* = 0.0001), stents (β = 0.362, *P* = 4 × 10^–27^), CK-MB (β = 0.076, *P* = 0.002), and hemoglobin (β = 0.200, *P* = 0.032) would increase total cost of hospitalization independent of age. Age (β = 0.487, *P* = 0.000001), serum sodium (β = 1.031, *P* = 5 × 10^–11^) and cTnI (β = 0.046, *P* = 0.022) would increase length of stay, while health insurance status (β = − 0.305, *P* = 0.0000001) and systolic blood pressure (β = − 0.334, *P* = 0.004) had the opposite effects. Age (β = 0.328, *P* = 0.045) and HDL-C (high density lipoprotein cholesterol, β = 0.390, *P* = 0.022) would increase nursing costs, while health insurance status (β = − 0.319, *P* = 0.004) had the opposite effects. Potassium (β = 0.513, *P* = 0.0003), stent (β = 0.394, *P* = 4 × 10^–23^) and health insurance status (β = 0.296, *P* = 0.0001) would increase daily hospitalization costs, while age (β = − 0.298, *P* = 0.009) had the opposite effects. HDL-C (β = 0.353, *P* = 0.007) and age (β = 0.321, *P* = 0.015) would increase daily nursing costs. Likewise, HDL-C (β = 0.360, *P* = 0.004), age (β = 0.538, *P* = 0.00001) increased the percent of nursing cost, while stent (β = − 0.332, *P* = 5 × 10^–13^) had the opposite effects (Table 3).

**Table 3:** Analyses of relevant factors by stepwise regression

	*β*	*P*	Adjusted R^2^
Total cost			
K	0.367	0.0001	0.785
Stent	0.362	4 × 10^–27^	
CK-MB	0.076	0.002	
Hb	0.200	0.032	
Length of stay			
Age	0.487	0.000001	0.849
Na	1.031	5 × 10^–11^	
Health insurance status	− 0.305	0.0000001	
SBP	− 0.334	0.004	
cTnI	0.046	0.022	
Nursing cost			
Age	0.328	0.045	0.186
Health insurance status	− 0.319	0.004	
HDL-C	0.390	0.022	
Daily cost			
K	0.513	0.0003	0.712
Stent	0.394	4 × 10^–23^	
Health insurance status	0.296	0.0001	
Age	− 0.298	0.009	
Daily nursing cost			
HDL-C	0.353	0.007	0.442
Age	0.321	0.015	
Percent of nursing cost			
HDL-C	0.360	0.004	0.516
Age	0.538	0.00001	
Stent	− 0.332	5 × 10^–13^	

Abbreviations as Table 1. He1alth insurance status: assign free medical care the value of 1, assign provincial medical insurance the value of 2, assign municipal urban employee medical insurance the value of 3, assign municipal urban resident medical insurance the value of 4, assign new rural cooperative medical system the value of 5, assign self-payment the value of 6

Health insurance status (β = − 0.186, OR = 0.830, 95% CI 0.694–0.993, *P* = 0.041) statins (β = − 1.118, OR = 0.327, 95% CI 0.191–0.560, *P* = 5 × 10^–5^), and serum sodium (β = − 0.04, OR = 0.961, 95% CI 0.945–0.977, *P* = 2 × 10^–6^) would decrease the occurrence of clinical events, age (β = 0.045, OR = 1.046, 95% CI 1.024–1.069, *P* = 4 × 10^–5^), heart rate (β = 0.034, OR = 1.035, 95% CI 1.019 − 1.052, *P* = 3 × 10^–5^) percent of nursing cost (β = 0.466, OR = 1.594, 95% CI 1.068–2.379, *P* = 0.022), and daily medication cost (β = 0.001, OR = 1.001, 95% CI 1.000 − 1.001, *P* = 0.007) had the opposite effects.

## Discussion

The rapid growth of medical and health expenditure has imposed a heavy burden on all countries in the world [1]. China’s medical and health expenditure increased from ¥1.45 trillion in 2008 to ¥4.10 trillion in 2015, with a compound annual growth rate of 15.96%. Moreover, the out-patient rate in China is lower than the world average, while the hospitalization rate is higher than the world average [7], which means that most of China’s medical expenses are spent on hospitalization. The actual cost of hospitalization depends on the health insurance status, spectrum of disease, kind of medical service received and the hospital visited.

In this study, hospitalized patients with acute myocardial infarction were selected, because acute myocardial infarction is a disease that must be hospitalized. Therefore, the influence of excessive hospitalization was excluded. For patients with the clinical presentation of STEMI (ST-elevated myocardial infarction) within 12 h of symptom onset and with persistent ST-segment elevation, early reperfusion therapy should be considered promptly, preferably using emergency coronary angiography with a view to primary PCI (percutaneous coronary intervention) or, if unavailable, intravenous thrombolysis. There is, however, no consensus as to whether PCI is also beneficial in patients presenting > 12 h from symptom onset in the absence of clinical and/or electrocardiographic evidence of ongoing ischaemia. Antithrombotic agents, β-blockers, angiotensin-converting enzyme inhibitor or angiotensin II receptor blockers, Mineralocorticoid receptor antagonist and statins should be given to all patients with acute myocardial infarction, exclusion of contraindications. Several recent studies have highlighted a fall in acute and long-term mortality following STEMI, in parallel with greater use of reperfusion therapy, primary percutaneous coronary intervention (primary PCI), modern antithrombotic therapy and secondary prevention treatments. However, the recommended therapies in these guidelines ignore the patient’s socio-economic status in real world. In China, medical insurance is closely related to social and economic status. Furthermore, most of medical insurance in China plays a role during hospitalization. Among different health insurance status, the age of free medical care is the largest, and the age of new rural cooperative medical system is the smallest. However, after controlling for age, there was no difference in disease indicators between the groups. This suggests that subsequent differences in cost and other indicators between groups may be due to age and different health insurance status.

In this study, 600 patients with acute myocardial infarction were selected to explore the influence of different medical insurance systems and nursing on the hospitalization cost and therapeutic effect. The purpose of our study is to explore the effect of different medical insurance on AMI patients during hospitalization. It is very likely that AMI patients would stay in hospital longer for fear of chest pain recurrence if they did not need to bear the expense. Different health insurance status has great differences in hospitalization costs [3]. Although there are differences in age among different health insurance status, after controlling for age, the hospital still spends the most at free medical care. Comorbidity due to age [8], increased reliance on nursing [9], the prolonged length of stay [10], may cause medication cost [8], the cost of the nurse, and longer duration of hospitalization. Comorbidity is very likely to have more drugs causing cost differences, and with the increase of age, drug cost differences may be large. The new rural cooperative medical system has the lowest age, then drug cost is small. After controlling for age, a greater difference between different health insurance status costs for medicine, nursing, and length of stay is still significant. Theoretically, hospitalization cost is closely related to aging [11]. However, this study has some similarities with previous studies on hospitalization rate [12], which found that population aging is not correlated with hospitalization. This suggests that health insurance status plays an important role in regulating the economics of hospitalization.

Further study on the efficiency of inpatient economic expenditure between different health insurance status found that daily inpatient expenses were affected by both health insurance status and age. Daily medication expenses are affected by health insurance status, while daily nursing expenses and percent of nursing expenses are affected by age. This means that the majority of hospitality-related costs are still driven by age and health insurance status. When improving the quality of care, the main lever for lowering costs is health insurance status.

Whether health insurance status affect therapeutic effect or not. After controlling for age, there was no difference in the disease indicators of each group, but occurrence of clinical events during hospitalization were affected by health insurance status. And the more the patients afford, the lower the risk of clinical events. This becomes an interesting paradox. The actual situation is that the more the patients afford, the younger the age is. Younger males may be more likely in higher income groups with a likely greater consumption of fat/sugars and more sedentary lifestyles than perhaps poorer and more rural patients. So young males would afford more medical cost. Furthermore, those with more co-payment are more concerned with therapeutic effect. Therefore, even the age is included, health insurance status still affects therapeutic effect. Age and health insurance status also affect length of stay. But for daily hospitalization costs, the lower the age, the poorer health insurance status, the lower the cost.

In clinical, medical treatment and nursing become a whole. Does nursing cost affect therapeutic effect? For the length of stay in hospital, the percent of nursing expenses had no effect. Since the older the age, the higher the dependence on nursing [9], total nursing cost, daily nursing cost, percent of nursing expenses that suffers an age to be big. Previous studies have shown that health insurance status affects patients’ choice between primary health care and nurse practitioners [13]. Our study confirmed that the worse the health insurance status, the lower the total nursing costs. Patients believe that drug treatment is more important than nursing, so those with worse health insurance status are more willing to pay for drug and operation other than nursing. But daily nursing costs and the percent of nursing costs were independent of health insurance status. The percent of nursing costs has certain influence on the occurrence of clinical events. The higher the percent is, the greater the probability of occurrence of events.

## Conclusion

In China, the hospitalization cost, length of stay and therapeutic effect of acute myocardial infarction patients are affected by different health insurance status and age. Nursing work is greatly affected by age, suggesting that older patients have higher dependence on nursing. The percent of nursing costs has a certain impact on the occurrence of clinical events.

## Data Availability

The datasets used and/or analyzed during the present study are available from the corresponding author on reasonable request.

## References

[1] Roth GA, Abate D, Abate KH (2018). GBD 2017 causes of death collaborators global, regional, and national age-sex-specific mortality for 282 causes of death in 195 countries and territories, 1980–2017: a systematic analysis for the global burden of disease study 2017. Lancet.

[2] Hu SS, Gao RL, Liu LS (2019). Summary of the 2018 report on cardiovascular diseases in China. Chin Circ J.

[3] Lin X, Cai M, Tao H, Liu E, Cheng Z, Xu C, Wang M, Xia S, Jiang T (2017). Insurance status, inhospital mortality and length of stay in hospitalised patients in Shanxi, China: a cross-sectional study. BMJ Open.

[4] Ludt S, Wensing M, Campbell SM, Ose D, van Lieshout J, Rochon J, Uhlmann L, Szecsenyi J (2014). The challenge of cardiovascular prevention in primary care: implications of a European observational study in 8928 patients at different risk levels. Eur J Prev Cardiol.

[5] Li M, Liu H (2018). Implementation of a clinical nursing pathway for percutaneous coronary intervention: a prospective study. Geriatr Nurs.

[6] Thygesen K, Alpert JS, Jaffe AS, Chaitman BR, Bax JJ, Morrow DA, White HD (2018). Executive group on behalf of the joint European society of cardiology (ESC)/American college of cardiology (ACC)/American heart association (AHA)/world heart federation (WHF) task force for the universal definition of myocardial infarction. Fourth universal definition of myocardial infarction (2018). Circulation.

[7] Moses MW, Pedroza P, Baral R, Bloom S, Brown J, Chapin A, Compton K, Eldrenkamp E, Fullman N, Mumford JE, Nandakumar V, Rosettie K, Sadat N, Shonka T, Flaxman A, Vos T, Murray CJL, Weaver MR (2019). Funding and services needed to achieve universal health coverage: applications of global, regional, and national estimates of utilisation of outpatient visits and inpatient admissions from 1990 to 2016, and unit costs from 1995 to 2016. Lancet Public Health.

[8] O'Connell J, Henman MC, Burke É, Donegan C, McCallion P, McCarron M, O'Dwyer M (2019). Association of drug burden index with grip strength, timed up and go and barthel index activities of daily living in older adults with intellectual disabilities: an observational cross-sectional study. BMC Geriatr.

[9] Kagan SH (2015). Editorial: ageing in place, older people and community nursing: gerontological nursing in place. Int J Older People Nurs.

[10] Ofori-Asenso R, Zomer E, Chin KL, Markey P, Si S, Ademi Z, Curtis AJ, Zoungas S, Liew D (2019). Prevalence and impact of non-cardiovascular comorbidities among older adults hospitalized for non-ST segment elevation acute coronary syndrome. Cardiovasc Diagn Ther.

[11] von Wyl V (2019). Proximity to death and health care expenditure increase revisited: a 15-year panel analysis of elderly persons. Health Econ Rev.

[12] Chandoevwit W, Phatchana P (2018). Inpatient care expenditure of the elderly with chronic diseases who use public health insurance: disparity in their last year of life. Soc Sci Med.

[13] Loresto FL, Jupiter D, Kuo YF (2017). Examining differences in characteristics between patients receiving primary care from nurse practitioners or physicians using medicare current beneficiary survey data and medicare claims data. J Am Assoc Nurse Pract.

